# Enhancing Bioluminescence Imaging of Cultured Tissue Explants Using Optical Telecompression

**DOI:** 10.3390/s24186041

**Published:** 2024-09-18

**Authors:** Jihwan Myung

**Affiliations:** 1 Braintime Laboratory, Graduate Institute of Mind, Brain and Consciousness (GIMBC), Taipei Medical University, New Taipei City 235, Taiwan; jihwan@tmu.edu.tw; 2 Graduate Institute of Medical Sciences, Taipei Medical University, Taipei 110, Taiwan

**Keywords:** bioluminescence microscopy, telecompression, relay lens optics, high numerical aperture optics, optical signal boosting, circadian clocks, suprachiasmatic nucleus

## Abstract

Long-term observation of single-cell oscillations within tissue networks is now possible by combining bioluminescence reporters with stable tissue explant culture techniques. This method is particularly effective in revealing the network dynamics in systems with slow oscillations, such as circadian clocks. However, the low intensity of luciferase-based bioluminescence requires signal amplification using specialized cameras (e.g., I-CCDs and EM-CCDs) and prolonged exposure times, increasing baseline noise and reducing temporal resolution. To address this limitation, we implemented a cost-effective optical enhancement technique called telecompression, first used in astrophotography and now commonly used in digital photography. By combining a high numerical aperture objective lens with a magnification-reducing relay lens, we significantly increased the collection efficiency of the bioluminescence signal without raising the baseline CCD noise. This method allows for shorter exposure times in time-lapse imaging, enhancing temporal resolution and enabling more precise period estimations. Our implementation demonstrates the feasibility of telecompression for enhancing bioluminescence imaging for the tissue-level network observation of circadian clocks.

## 1. Introduction

Real-time observation of dynamic cellular processes within living tissues is crucial for understanding biological phenomena at the network level. Synaptic and diffusive signaling among cells on timescales of milliseconds to minutes trigger intracellular signaling cascades, causing changes in gene expression patterns in individual cells. These patterns reflect the structure of a local connectome, the network of connections among cells. Circadian clocks are dynamic oscillations in the expression of clock genes. Observing gene expression in circadian clocks provides an ideal system for probing network dynamics. It has been computationally demonstrated that the short timescale dynamics of intercellular communication can shape the spatial patterning of circadian gene expression in the suprachiasmatic nucleus (SCN), the master pacemaker tissue that regulates the circadian rhythms of the rest of the body [1].

Organotypic culture based on a semi-permeable membrane enables a stable culture and long-term observation [2]. By culturing tissues expressing reporters on membranes under the microscope, we can now track the evolving gene expression patterns. This method allows us to decipher the long-term functional connectome and reveal how the network coordinates cellular behaviors, such as the timing of the circadian clock in each cell [3].

Reporter gene expression enables real-time observation of live systems by inserting a coding sequence that produces a detectable signal when a gene of interest is transcribed. Green fluorescent protein (GFP) and its variants are commonly used for optical measurement of these activities. This fluorescent reporter technique is particularly effective for revealing short-term dynamics and has been highly successful in monitoring calcium dynamics, as demonstrated with GCaMP [4]. However, fluorescent reporters are often not suitable for long-term culture imaging due to phototoxicity caused by the ultraviolet (UV) light required for excitation. This can damage cells and the reporter molecule itself. Additionally, fluorescence has a limited dynamic range and often produces nonspecific background fluorescence that can obscure weak signals.

In contrast, bioluminescence reporters, such as luciferase, offer a distinct advantage for long-term observation. Firefly luciferase (Fluc), for example, was first adopted as a reporter of gene transcription, analogous to the β-galactosidase assay. The luciferase assay does not require histological processing, such as fixation, and live samples can be used as is for measurements. This technique relies on the bioluminescence reaction involving luciferase, luciferin, and ATP, which produces light through an enzyme-catalyzed process. In this reaction, luciferase catalyzes the oxidation of luciferin in the presence of ATP and oxygen, resulting in the formation of oxyluciferin. As oxyluciferin returns to its ground state, the excess energy is released as photons, producing visible light. Since luciferin is membrane-permeable, it can be added to the culture medium to generate light without the need for excitation light, unlike fluorescence-based reporters. The low light signal generated by luciferase can be difficult to capture without sensitive light detectors, such as the photomultiplier tubes used in luminometers. Nonetheless, while the signal and noise of fluorescent reporter detection are influenced by excitation and emission, the detection of bioluminescence reporter activity is primarily limited by the background noise of the detection apparatus [5]. Bioluminescence does not require external excitation since light is produced within the cell as long as the substrate is present [6]. This eliminates phototoxicity concerns and allows for continuous, long-term monitoring.

It should be noted that despite the low brightness of the signal, a single luciferase molecule, such as Fluc, produces multiple photons. This makes the absolute quantification of the number of reporter molecules difficult, though not impossible [7]. The accepted unit for bioluminescence is therefore the relative light unit (RLU). Here, since we base the measurements on the brightness registered and digitized by the same charge-coupled device (CCD), we report bioluminescence values in terms of 16-bit pixel intensity values.

Advancements in CCDs have revolutionized the use of bioluminescent reporters for visualizing spatial gene expression, both in vitro and in vivo [8]. The organotypic culture of the SCN demonstrated the powerful potential of bioluminescence reporter systems for systems biology, particularly in studying synchronization dynamics among circadian clock neurons at the intratissue network level [9]. Since then, reporter imaging methods have been extensively employed to reveal network dynamics beyond the circadian timescale, including photoperiodic encoding in tissue explants [10]. The success of bioluminescence imaging in circadian biology has led to computational investigations into dynamic network interactions [3].

However, bioluminescence signals are inherently weak, posing a significant challenge for imaging, particularly at the tissue level where a large field of view and typically low-magnification objectives are required, which gather less light. Efforts to enhance bioluminescence imaging have focused on two key strategies: developing brighter luciferases and utilizing high-sensitivity cameras. Brighter luciferases, such as the emerald luciferase (ELuc) from the Brazilian click beetle, increase overall signal intensity [11]. Alternatively, high-gain cameras like intensified CCDs (I-CCDs) and electron-multiplying CCDs (EM-CCDs) amplify the weak signal. Nevertheless, these approaches have limitations. Brighter luciferases can lead to a higher baseline signal, potentially reducing the signal-to-noise ratio (SNR) [12]. EM-CCDs, while amplifying the signal, also amplify the inherent dark current noise within the camera. This can limit the overall image quality, and EM-CCDs are significantly more costly than conventional cooled CCDs [13].

In popular photography and astronomical imaging, there is a technique that increases the light-gathering ability of the lens, known as telecompression, or more popularly, the “speed booster” [14]. It is an adapter that sits between the sensor and the lens, reducing the lens’s magnification. This is the opposite of a teleconverter, which increases magnification. Higher magnification (telephoto) lenses collect light from a narrower field of view, concentrating the light onto a smaller area. A speed booster can then expand this field of view. Importantly, it does so while preserving the total number of photons collected. A similar principle can be directly applied to bioluminescence imaging. By using a high-magnification and high-numerical-aperture objective along with a low-magnification converting relay lens between the objective and the CCD, more photons can be gathered per single cell that emits bioluminescence.

Telecompression can provide a cost-effective solution to optical enhancement of bioluminescence imaging, especially because the lack of excitation–emission simplifies the optics. We employed this technique in our first timelapse bioluminescence imaging about a decade ago, where we revealed that single-cell oscillation periods in the SCN can differ under explant culture conditions [12]. However, we did not focus on this optical method or rigorously quantify the signal improvements achieved through telecompression. Here, we compare the signal improvements over conventional optics to illustrate its clear benefits.

## 2. Materials and Methods

### 2.1. Brain Slice Culture

Heterozygous reporter mice expressing PER2::LUC (*n* = 4, aged 3–4 weeks; Jackson Laboratory) were sacrificed under anesthesia, and their brains were rapidly isolated and transferred to ice-cold Hank’s Balanced Salt Solution (HBSS), as described previously [12]. Coronal hypothalamic slices (250 µm thickness) were prepared using a Vibratome and trimmed to isolate the SCN region. Each slice was placed on a PTFE culture membrane with pore size 0.4 μm (PICM0RG50, Millipore, Bedford, MA, USA) in a 35 mm dish (Corning, NY, USA) containing 1 mL of phenol red-free Dulbecco’s Modified Eagle Medium (DMEM; Sigma, St. Louis, MO, USA) with B27 Supplement (Gibco, NY, USA), penicillin-streptomycin (0.25×, Gibco), and 100 µM beetle luciferin (Promega, Madison, WI, USA). The dish was sealed with vacuum grease (Corning) to prevent evaporation and maintained in a stage-top incubator during observation. All procedures were in line with protocol LAC2023-0038, approved by the Institutional Animal Care and Use Committee of Taipei Medical University.

### 2.2. Objective Lenses and Relay Lens

We evaluated a combination of a high numerical aperture objective lens with a relay lens on a microscope frame housed in a light-sealed dark box: CFI Plan Fluor 10×/N.A.0.30 W.D. 16 mm (Nikon, Japan) (abbreviated as 10×) and L Plan EPI 20×/N.A.0.45 W.D. 10 mm (Nikon) (20×) with a 0.55× reducing relay lens (C-0.55DS, Nikon) (20× + 0.55). For an additional comparison of the numerical aperture at the same magnification, we used a CFI Plan 10×/N.A.0.25 W.D. 10.5 mm objective (10×/0.25). For a demonstration of time-lapse imaging, we used an LUCPLFL N 40× NA0.6 (Olympus, Japan) objective lens, combined with a U-TV0.25XC relay lens and a U-TLU (Olympus) tube lens. While the relay lens is susceptible to internal reflection (flare) at high illumination levels, this issue did not occur at the low light levels used in bioluminescence imaging. The upright configuration required a minimum working distance of 10 mm, corresponding to the thickness of a standard 35 mm culture dish. All objective lenses met this criterion, allowing for continuous recording for approximately one week without changing the culture medium.

### 2.3. Considerations for Temporal Variability in Circadian Intensity Imaging

Since the PER2::LUC intensity changes over the circadian cycle in SCN explants, we minimized the recording duration for each sample to reduce variation due to the time of recording. This led us to choose a 5 min exposure time with an 8-binning setting. Under this setting, for three of the four samples, the time difference between image captures using the 10× and 20× + 0.55 configurations was within 10 min. One sample took longer due to detailed imaging trials at different settings.

### 2.4. Image Acquisition and Processing

Images were acquired using a cooled CCD camera (Orca R2, C10600-10B, Hamamatsu, Japan) with 8-binning and a 5 min exposure for tissue-level signal comparison. For cellular-level comparison, 4-binning and a 15 min exposure were used. The camera featured a 2/3-inch interline CCD with a peak quantum efficiency of 70% at 500 nm, extending to 600 nm in low light mode. The C-0.55DS relay lens exhibited a peak transmittance of 98% at 570 nm and maintained 97% within a ±100 nm range. This system was optimized for the firefly luciferase emission spectra (560 nm) and its variants (500–650 nm) [8,10]. Image acquisition was controlled by MicroManager 1.3.43 (based on ImageJ 1.43j, University of California San Francisco, CA, USA and NIH, Bethesda, MD, USA) using the Hamamatsu DCAM driver. Images were stored as 16-bit grayscale TIFFs, and noise was removed using ImageJ’s Remove Outliers function. Although the Orca R2 camera exports data in 16-bit digital format, it uses a 12-bit in-camera analog-to-digital converter, limiting the dynamic range of the quantized luminescence to 0–4095, rather than the full 0–65,535 available in 16-bit TIFFs. Since the dynamic range of the LCD display is limited to 8 bits (0–255) or 10 bits (0–1023), the acquired raw images appear black on the display. To make the images visible and comparable, we set the display range to 1600–2600 in all presentations.

For single-cell analysis using ImageJ, square regions of interest (ROIs) of uniform size (~11 × 11 µm) were selected to encompass the bioluminescence pattern of a single SCN cell (typical diameter ~10 µm, with the slightly larger area accounting for minor cell movements during ex vivo culture, which were mostly contained within the ROI grid [12]. ROIs were chosen to avoid immediate cell aggregates, and the area outside each cell was mostly dark. The positional jitter of the cells within the ROIs was estimated by their deviation from their original position over the culture period, quantified by the Euclidean distance of the center of mass (∆CM) at time *t*:

$$∆\mathrm{CM}(t)=\sqrt{{\left(x_{CM}(t)-x_{CM}\left(0\right)\right)}^{2}+{\left(y_{CM}(t)-y_{CM}\left(0\right)\right)}^{2}}$$

which averaged less than one pixel over 7 days (Supplementary Figure S2B). The average intensity values from the ROIs were exported for further analysis in Mathematica (Wolfram Research, Champaign, IL, USA).

### 2.5. Statistical Analysis

For the tissue-level comparison of signals under different optical configurations, statistical significance was determined using Student’s *t*-tests in Mathematica (Wolfram Research). For single-cell region of interest (ROI)-level comparisons, due to unequal sample sizes, the Mann–Whitney *U* test was used. A *p*-value of less than 0.05 was considered significant.

## 3. Results

### 3.1. Image Brightness and Numerical Aperture

**Figure 1:**
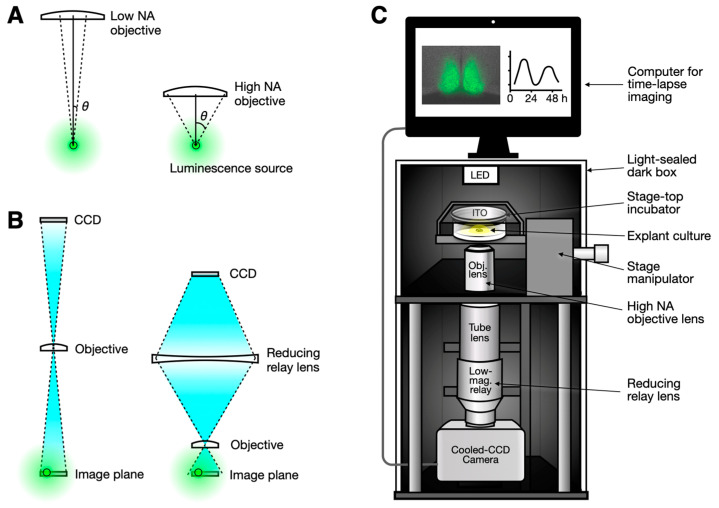
Optical configurations for enhancing bioluminescence imaging. (**A**) A high-numerical aperture (NA) objective collects more light due to its large acceptance angle (*θ*). (**B**) A low-magnification objective lens typically has a low NA (**left**). While a high-NA objective lens is usually associated with high magnification, this can be compensated for by adding a reducing relay lens, thereby preserving the high photon collection capability of the high-NA objective (**right**). (**C**) A simple microscopy setup is proposed. A combination of a high-NA objective and a reducing relay lens can be easily implemented on an open frame or on available commercial microscope frames, whether in upright or inverted configurations. The camera can be a conventional cooled CCD camera, with the entire setup housed in a light-sealed dark box for bioluminescence imaging.

The brightness of the image, or the signal (*S*) detected by the CCD, depends on both the numerical aperture (NA) and the magnification (*M*). The numerical aperture is a measure of the objective lens’s ability to collect light. A bioluminescent source, such as cells expressing luciferin, emits photons in all directions. A high-NA objective lens has a wider acceptance angle, allowing it to capture more light (Figure 1A). The NA is proportional to the sine of the acceptance angle (*θ*), which is proportional to the radius of the image circle. Consequently, the amount of light collected is proportional to the square of the radius, and thus to the square of the NA.

Conversely, higher magnification results in only a portion of the image circle being projected onto the imaging sensor (e.g., CCD), leading to reduced brightness. Since the area of the sensor is inversely proportional to the square of the magnification factor, the brightness is inversely proportional to the square of the magnification. Therefore, the approximate relationship between signal strength, numerical aperture, and magnification can be expressed as follows (Equation (1)) [15]:

*S* ~ NA^2^/*M*^2^ (1)

The per-pixel signal maximization in bioluminescence imaging is determined by the following equation [16]:

*S* = (Photons emitted from a source) × (Collection efficiency) × (Detection efficiency) (2)

Our approach improves collection efficiency by combining a high-NA objective with a demagnifying relay lens. The relay lens compresses the image circle from the high-NA objective, fitting it onto the CCD sensor while preserving the total photon count (Figure 1B). Since the high-NA objective gathers more photons, combining it with a demagnifying relay lens maximizes photon collection and offsets the efficiency loss from the objective’s high magnification.

In practical implementations, there are additional considerations when using relay lenses. If a simple lens is used, chromatic aberrations can be introduced. However, this may be less of a concern in monochromatic luciferase systems, where only a single-peaked wavelength of light is emitted (emission range between 540 and 580 nm, with a peak at 560 nm in firefly luciferase) [17]. If a complex lens is used, the optical diameter can be limited. The relay lens diameter should, however, exceed the tube lens diameter to prevent vignetting.

An accessible and affordable setup for bioluminescence imaging can be created based on these principles (Figure 1C). Since bioluminescent reporters do not require excitation or brightfield illumination, the optical system can be greatly simplified. The objective lens can be directly connected to the tube lens, as most modern objective lenses use infinity-corrected optics. A reducing relay lens is then placed between the tube lens and the CCD camera. This configuration has the additional benefit of shortening the light path length and maintaining high collection efficiency. For focusing, ambient light from the environment is usually sufficient, though an LED can be added directly above the sample without a condenser or positioned on the side for a slight oblique illumination effect [18]. This setup can be housed in a dark box to measure bioluminescence, with the LED turned off.

### 3.2. Testing in the Suprachiasmatic Nucleus Expressing PER2::LUC

To test the concept, we used mouse SCN explants expressing the canonical bioluminescent reporter PER2::LUC [19]. The SCN tissue has physical dimensions that fit within a 500 µm diameter. With the 2/3-inch sensor in our cooled CCD camera (Orca R2, Hamamatsu), a 10× objective provides the ideal magnification for capturing the entire coronal SCN slice. We thus compared a 10× objective (NA 0.30) with a higher NA 20× objective (NA 0.45) combined with a 0.55× relay lens, resulting in an effective magnification of 11×. The sensor diameter (16.9 mm) is smaller than the nominal diameter of the C-mount (25 mm) coupling system, and the service port has a typical diameter of 40 mm. Thus, there is ample room for image circle compression through the reducing relay lens.

**Figure 2:**
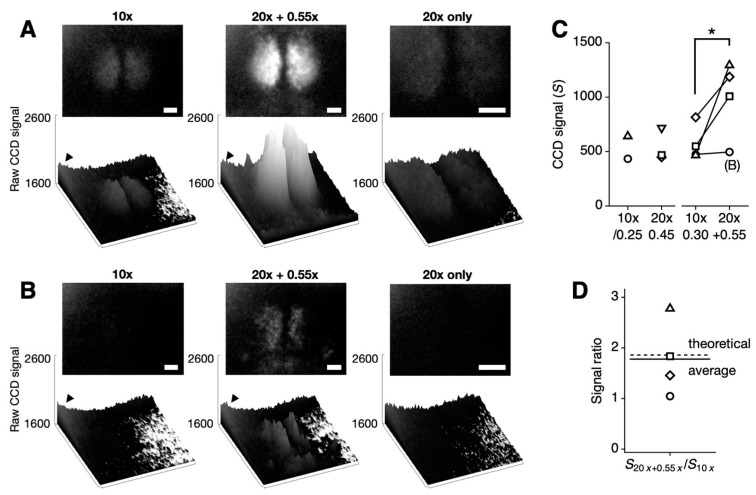
Comparison of bioluminescence imaging with standard and enhanced optical configurations in SCN slices. (**A**,**B**) Representative bioluminescence images collected using a Plan Fluor 10×/0.30 objective (10×) (**left**) compared with an L Plan 20×/0.45 objective combined with a 0.55DS relay lens (20× + 0.55×), resulting in an effective magnification of 11× (**middle**), and without a relay lens (20× only) (**right**). The upper images are 16-bit TIFFs with CCD signal values consistently presented between 1600 and 2600. The lower images show the corresponding CCD signals as 3D bar graphs. Note the systematically higher baseline noise in the upper-left corner (indicated by an arrowhead). White scale bars represent 100 µm. (**B**) The bioluminescence signal, weaker than the baseline noise level, is not visible on the CCD (**left**) but becomes detectable with the enhanced optical setup (**middle**), while it remains undetectable when only a 20x objective is used (**right**). (**C**) CCD signal values were calculated by subtracting the baseline noise from the raw signal. The single-pixel signal does not increase significantly with the slight increase in NA (10×/0.25 vs. 10×/0.30). No improvement in signal intensity was observed when only a 20× objective was used (*p* = 0.09, paired Student’s *t* test, *n* = 3 slices). However, signal intensity significantly increased with the higher NA objective and reducing relay lens combination (10×/0.30 vs. 20×/0.45 + 0.55×; * *p* < 0.05, paired Student’s *t* test, *n* = 4 slices). The open circles indicate the low bioluminescent sample shown in (**B**), which was not accurately quantified due to the presence of systematic noise. See Supplementary Figure S1 for images taken with the 10×/0.25 objective. (**D**) The signal ratio between the 20× + 0.55× combination and the 10× objective is 1.78-fold (mean of 4 slices, solid line), closely matching the theoretical value of 1.86-fold (dashed line).

We analyzed tissue-level bioluminescence signals in four SCN slices per condition. Signal intensity was quantified as the difference between the maximum and minimum raw signal values (ranging from 0 to 65,535 in 16-bit format). Representative bioluminescent images for each optical configuration are shown in Figure 2A,B (from different mice). The baseline profile was consistent across all images, regardless of the optics used, reflecting the characteristics of the CCD sensor. A higher baseline in the upper-left corner (indicated by arrowhead) is a known characteristic of this sensor [12], likely due to the Peltier cooling system.

As shown in Figure 2, the combination of a high-NA objective and a reducing relay lens significantly enhanced the signal. Signal intensity increased across all four SCN slices tested (* *p* < 0.05; 10× objective: 576.0 ± 81.8 vs. 20× with 0.55× relay lens: 996.3 ± 176.9; mean ± SEM, *n* = 4). Although the signal increase was evident in most cases, it was masked by higher baseline noise in the upper-left corner of the sample shown in Figure 2B (indicated as (B) in Figure 2C). The theoretically predicted signal improvement was 1.86-fold, while the measured average showed a 1.78-fold increase (Figure 2D). This increase in signal without a corresponding increase in baseline noise demonstrates that our method specifically improves collection efficiency without affecting photon count or detection efficiency (Equation (2)). With a modest increase in NA from 0.30 to 0.45, this represents a significant per-pixel increase in signal strength.

### 3.3. Enhanced Bioluminescence Signal Detection at Single-Cell Resolution

**Figure 3:**
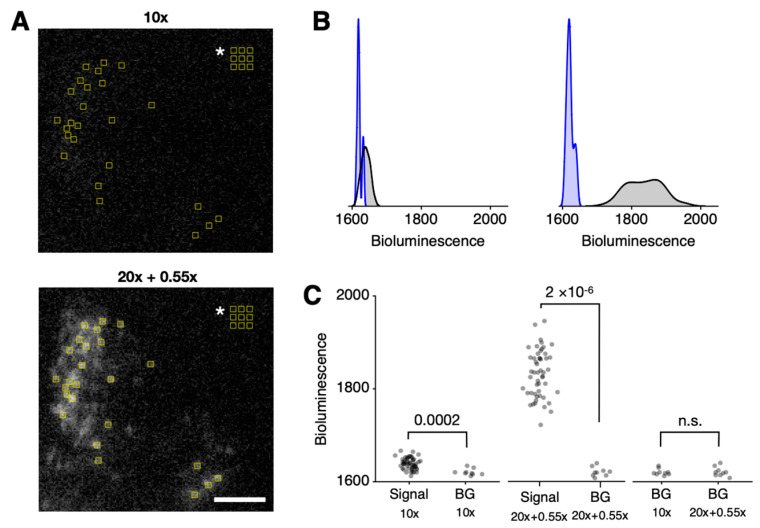
Detailed single-cell-level analysis of bioluminescence. (**A**) Single-cell-sized ROIs are selected in the positions of cells and background (indicated by white asterisk in the upper right corner) for bioluminescence quantification. A half-hemisphere of the SCN is presented. The white scale bar represents 100 µm. (**B**) In the histograms of quantified bioluminescence values, the background distribution (blue) is distinguishable from the signal distribution (black) in both the 10× (**left**) and 20× + 0.55× (**right**) settings. (**C**) In the raw bioluminescence scatterplot of each ROI, the difference between the signal and background (BG) groups is statistically significant for both 10× (far left; *p* = 0.0002; *n* = 51 for Signal, *n* = 9 for BG) and 20× + 0.55× (middle; *p* = 2 × 10^−6^; *n* = 51 for Signal, *n* = 9 for BG). For the background values, there was no statistically significant difference (far right; *p* = 0.89; *n* = 9 in each group). Mann–Whitney *U* test. See Supplementary Figure S2 for circadian timelapse images and ROI analysis.

We examined whether the gain in bioluminescence signal at the tissue level translates to single-cell resolution against the background, where baseline noise is dominant. Under 4-binning, single cells could be resolved with a 15 min exposure. Analysis of single-cell-sized regions of interest (ROIs) revealed that the telecompression method enables the reliable measurement of bioluminescence in single cells (Figure 3A).

Although bioluminescence signals from single cells were statistically distinguishable from the background in both optical setups using the standard *p*-value criterion, a clear difference was observed in all ROIs from recordings made with the telecompression setup. None of the ROIs from cells showed bioluminescence values overlapping with the background under the telecompression (20× + 0.55×) configuration, whereas some ROIs overlapped with the baseline in the 10× configuration (Figure 3B). Consistent with the principles of telecompression, which only enhance light collection, the background luminescence remained consistent across optical setups (Figure 3C, far right).

## 4. Discussion

We demonstrated a straightforward optical configuration that enhances signal strength in bioluminescence imaging. The microscopy setup proposed in Figure 1C can be easily constructed using commercially available components or adapted to existing microscopy setups. In this demonstration, we used Nikon components, but we have also tested similar configurations with Olympus, Leica, Mitutoyo, and generic components, all yielding comparable improvements in signal strength. It is evident that this simple and economical construction provides clear benefits in increasing the collection efficiency of bioluminescence signals. Although this optical improvement can only be achieved with a high-NA objective lens, high-NA lenses at low magnification are often highly expensive or even unavailable. In our example, we boosted an NA of 0.45 at an effective magnification of 11×; however, it can be increased to NA 0.60 by combining a 40x objective lens with a 0.25× relay lens (Supplementary Figure S2). Therefore, applying the telecompression principle to bioluminescence imaging offers clear advantages.

One key advantage of the enhanced light collection for circadian systems is the reduction of exposure time, which shortens the sampling interval in timelapse imaging. Circadian clocks in different parts of the body express characteristic period lengths [19]. Even within the SCN tissue, subtle differences in period length can exist among circadian clock cells when freerunning under explant culture [12]. Resolving these subtle differences using spectral methods like Fast Fourier Transform can be challenging when the sampling interval is limited to the standard 1 h. However, our method allows for single-cell resolution with a 15 min exposure under 4-binning (Figure 3). This significantly boosts the spectral resolution of single-cell circadian oscillations in conventional cooled CCD cameras.

However, the telecompression approach has its limitations. As shown in the low bioluminescent sample (Figure 2B), the bioluminescent signal must exceed the CCD’s baseline noise level for detection. While our approach can partially address this issue, signal strength can be further improved by engineering the luciferase for higher brightness [11]. Despite this, the telecompression method offers the advantage of enhancing the signal while keeping baseline noise unchanged. On the other hand, we found that EM-CCDs amplify noise during long exposures, whereas bioluminescence images obtained using conventional cooled CCDs with the telecompression system were less noisy. Conventional CMOS (Complementary Metal–Oxide–Semiconductor) sensors have not been optimal for bioluminescence imaging due to their lower signal-to-noise ratio and limited exposure time compared to CCD sensors. The cooled scientific CMOS (sCMOS) sensors we tested did not suppress baseline noise during long exposures as effectively as cooled CCDs (see data from the Hamamatsu ORCA-Quest qCMOS in Supplementary Figure S3. In comparison, a more conventional Sony IMX183-based imager did not return any bioluminescence image). While sCMOS sensors provide excellent images for fluorescence imaging due to their much lower read noise, their greater dark noise during long exposures makes them less suitable for bioluminescence imaging compared to CCDs. There have been recent developments in CMOS sensor technology, including improved quantum efficiency through back-illumination, reduced readout noise, and increased full well capacity, although exposure time remains limited. Using the latest engineered luciferase, NanoLuc (100-fold brighter than firefly luciferase), along with a qCMOS camera, volumetric bioluminescence imaging in *Caenorhabditis elegans* was recently demonstrated through computational image restoration using deep learning [20]. With parallel advancements in emission, collection, and detection (Equation (2)), bioluminescence shows great promise as the next reporter of choice for monitoring tissue culture systems, including organoids.

The telecompression system has another limitation when high magnification images are required. To utilize a reducing relay lens effectively, a higher magnification objective is needed, often requiring oil or water immersion beyond 40x magnification. We found that maintaining imaging with immersion objectives is impractical. Water immersion lenses are problematic due to contamination and issues with temperature and moisture control. Oil immersion lenses, on the other hand, often have a working distance too short to reach the tissue explant sample. Additionally, with the high temperatures maintained in the stage-top incubator, the immersion oil dries out over the course of the observation period, which typically lasts a week for circadian experiments. Therefore, the relay lens solution is the most suitable for effective magnification at around 10×, which is ideal for visualizing and analyzing network dynamics in cultured systems.

With the development of red-shifted luciferase substrates, such as CycLuc1 (emission wavelength 599 nm) and TokeOni (675 nm), in vivo tissue imaging of bioluminescent reporter activity is becoming more feasible [21]. Although achieving near-infrared emission without mutations in luciferase remains challenging, these new approaches open up opportunities for deeper tissue imaging in live animals, especially with the development of the skin-transparenting technique using an orange–yellow dye, which allows for the transmission of these long-wavelength signals [22]. Since telecompression increases the field of view, the method may also be useful for in vivo imaging, although additional considerations are required for low-wavelength optics.

Using telecompression, a technique originally used in other fields of digital photography, we constructed our system to monitor tissue-wide gene expression activities at a low cost. This method is particularly well-suited for time-lapse imaging of circadian clock activities. However, there are instances where high spatial resolution imaging of luciferase reporter expression is needed. For these purposes, adopting a technique from astrophotography known as “lucky imaging” could offer improvements [23]. As discussed above, CMOS sensors are not advantageous for long exposures, but they can be used in lucky imaging to take multiple short exposure images, selecting and collating those that meet an SNR standard. However, time-lapse imaging requires the quantification of the signal intensity (which essentially means photon counting) over a specific exposure interval, making lucky imaging unsuitable for monitoring circadian oscillations. Nevertheless, we note that exploring methods from other fields holds great promise for advancing bioluminescence imaging techniques.

## 5. Conclusions

Our telecompression-based optical configuration offers a simple and cost-effective solution for enhancing bioluminescence imaging, particularly for timelapse studies of circadian rhythms. By reducing exposure times, the method can reveal subtle differences in circadian periods at the single-cell level. While our approach has limitations, the potential for further improvements continues to show the promise of bioluminescence reporters as powerful tools for monitoring cellular activities in organ cultures. 

## Data Availability

The processed bioluminescence data used for this study are available from the corresponding author upon reasonable request. Requests should include a brief description of the intended use of the data.

## Supplementary Materials

The following supporting information can be downloaded at: https://www.mdpi.com/article/10.3390/s24186041/s1. Figure S1: SCN explant PER2::LUC images captured with 10×/0.25, 10×/0.30, 20×/0.45, and 20×/0.45 combined with a 0.55× relay lens. Figure S2: Circadian timelapse imaging of PER2::LUC bioluminescence signals from the cultured posterior SCN (pSCN) explant. Figure S3: SCN PER2::LUC imaging through a quantitative CMOS (qCMOS) sensor.
