# Supplementary material for: Enhancing Bioluminescence Imaging of Cultured Tissue Explants Using Optical Telecompression
Source: Sensors (Basel). 2024 Sep 18;24(18):6041. doi: 10.3390/s24186041 (PMC11436007; doi:10.3390/s24186041)
Supplement: Supplementary file 1 [file sensors-24-06041-s001.zip › sensors-3192698-supplementary.pdf]

Supplementary Figures:

Enhancing Bioluminescence Imaging of Cultured Tissue Explants using Optical Telecompression

Jihwan Myung [1,2,*]

[1] Braintime Laboratory, Graduate Institute of Mind, Brain and Consciousness (GIMBC), Taipei Medical University, New Taipei City 235, Taiwan
[2] Graduate Institute of Medical Sciences, Taipei Medical University, Taipei 110, Taiwan
* Correspondence: jihwan@tmu.edu.tw

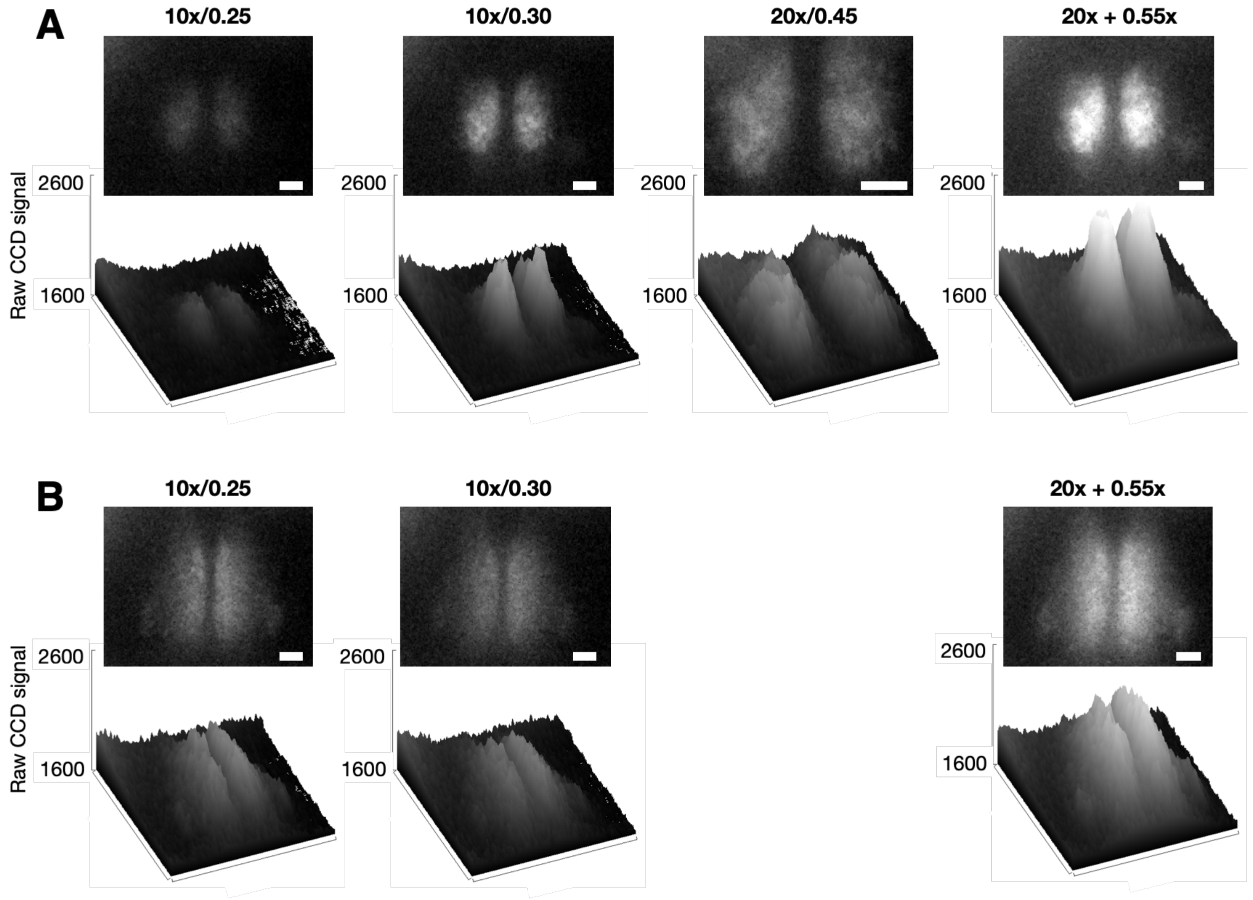

Supplementary Figure S1. SCN explant PER2::LUC images captured with 10x/0.25, 10x/0.30, 20x/0.45, and 20x/0.45 combined with a 0.55x relay lens.

These are raw data corresponding to Figure 2C for two different samples, (**A**) and (**B**), comparing CCD signal intensity across different objective and relay lens combinations. All images are presented for the range of 1600 to 2600.

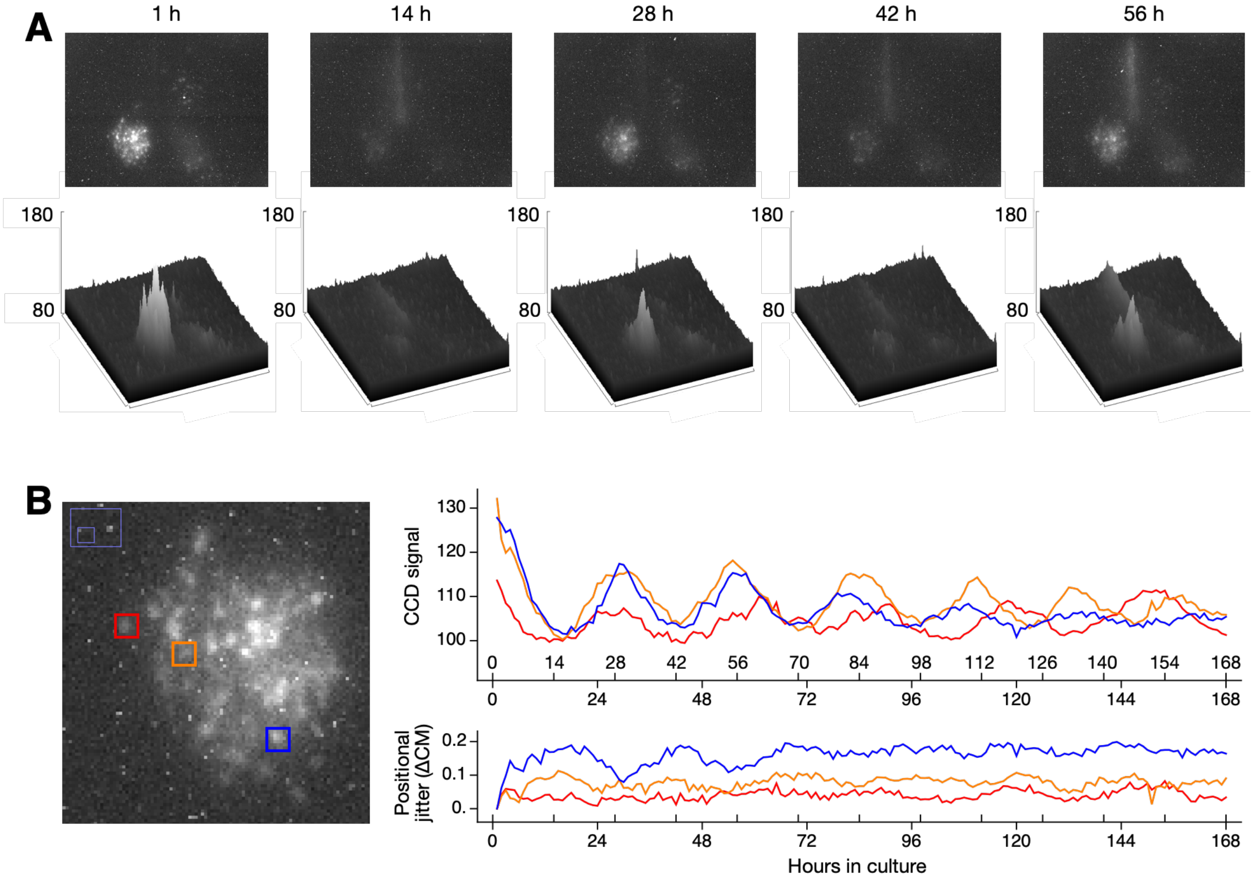

Supplementary Figure S2. Circadian timelapse imaging of PER2::LUC bioluminescence signals from the cultured posterior SCN (pSCN) explant.

(**A**) Timelapse images of the pSCN explant cultured for 7 days (168 hours). Images are shown for the range of 80 to 180, obtained using a Hamamatsu Orca R2 cooled CCD camera with an Olympus LUCPLFL N 40X NA0.6 objective lens combined with a 0.25x relay lens. The bioluminescence signal increases and decreases with a near 28-hour periodicity. (**B**) Three cells (red, orange, blue) are selected with a square ROI (left). The rectangles in the upper-left corner indicate the area shown relative to the full CCD image. The three cells within their respective ROIs display circadian oscillations, initially synchronized, but gradually detuning over the course of culture time (upper right). The jitter of the center of mass is confined within a single pixel magnitude throughout the culture duration, indicating that in this tissue culture system, the ROI selection is stable (lower right).

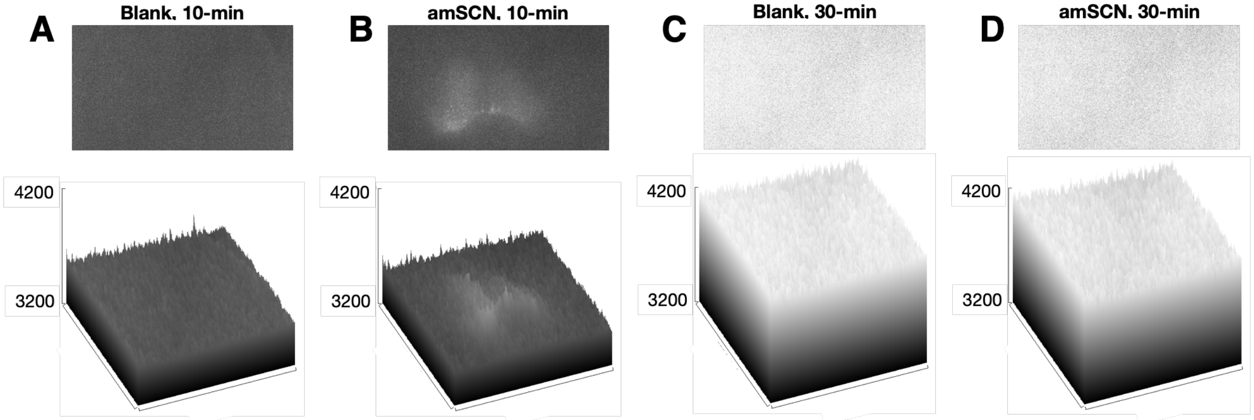

Supplementary Figure S3. SCN PER2::LUC imaging through a quantitative CMOS (qCMOS) sensor.

Compared to the baseline noise in the cooled CCD (Supplementary Figure 2; from the posterior SCN of the same animal), the CMOS sensor shows higher baseline noise as the exposure time increases. (**A**) 10-minute exposure without sample (blank). (**B**) 10-minute exposure with an anterior-mid section of the SCN (amSCN). (**C**) 30-minute exposure without sample. (**D**) 30-minute exposure with amSCN. Images are shown for the range of 3200 to 4200. Imaging was performed using a Hamamatsu ORCA-Quest cooled qCMOS camera (4x binning) with an Olympus LUCPlanFL N 20X NA0.45 objective lens combined with a 0.5x relay lens.
